# Cardiovascular magnetic resonance of the charcoal heart

**DOI:** 10.1186/1532-429X-10-37

**Published:** 2008-07-09

**Authors:** Vikas K Rathi, Ronald B Williams, June Yamrozik, Howard Grill, Robert WW Biederman

**Affiliations:** 1 Division of Cardiology, Allegheny General Hospital, Drexel University College of Medicine, 320 E North Avenue, Pittsburgh, PA-15212, USA

## Abstract

We report a case of malignant melanoma metastasis to the heart presenting as complete heart block. The highlight of the case is to demonstrate that silent cardiac metastasis is not uncommon and CMR has the potential to characterize these cardiac metastases and should be used routinely as a screening tool for those cancers with a high chance of cardiac involvement.

## Background

Metastasis to cardiac structures is rare. The common tumors that metastasize to the heart are breast cancer (direct extension), malignant melanoma (hematogenous), lung carcinoma (venous extension, direct invasion) and hypernephroma (venous extension) [1,2]. Cardiac metastasis of malignant melanoma bears poor prognosis, especially as it may not produce any symptoms due to the indolent nature of the metastasis. We present a case of malignant melanoma metastasis to the heart, incidentally diagnosed due to bradycardia and complete heart block detected on a routine evaluation.

## Case presentation

**Figure 1:**
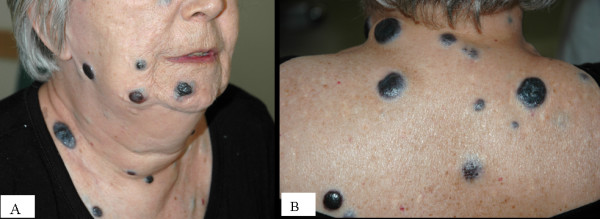
Photograph of the face (A) and the upper back (B) demonstrate large nodular melanoma deposits on the skin.

**Figure 2:**
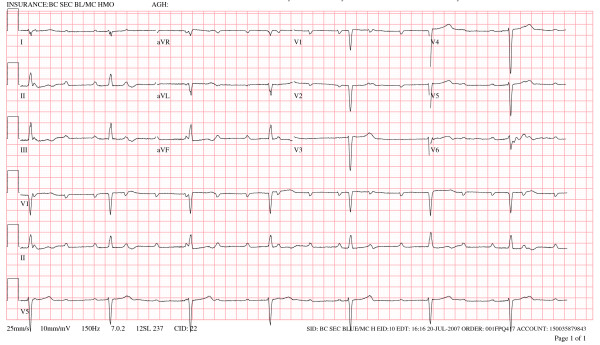
Twelve lead ECG demonstrating sinus tachycardia with complete heart block and slow ventricular junctional escape rhythm.

**Figure 3:**
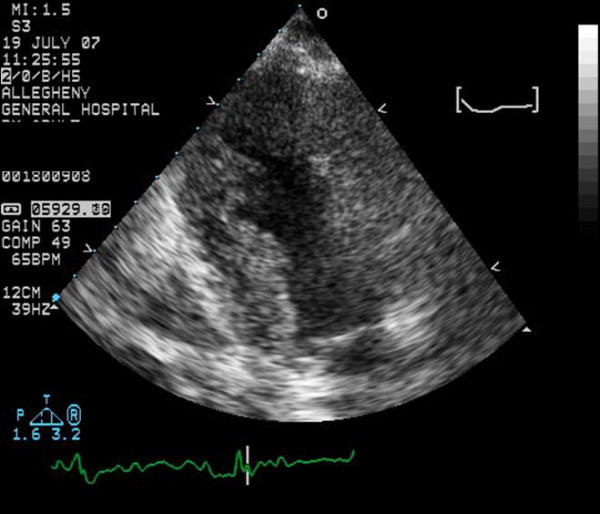
A two chamber view of the transthoracic echocardiogram demonstrating myocardial thickening and slightly irregular endocardial margins. There are no nodular deposits seen on this image.

**Figure 4:**
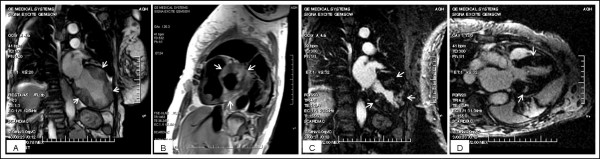
A) SSFP cine still frame demonstrates nodular appearance of the myocardium (arrows) with slightly bright signal compared to normal myocardium. Also, the endocardial border is irregular. B) T2-weighted image demonstrating bright nodular melanoma deposits (arrows) within the myocardium. C) 5 min post-contrast there is extensive patchy but discrete areas of myocardial enhancement (arrows) correlating with the nodular appearance of myocardium in 4A. D) left ventricular outflow tract view demonstrating patchy enhancement in the interventricular septum and posterior wall (arrows).

A 67 year old Caucasian female with a past medical history significant for cutaneous malignant melanoma diagnosed 7 years ago with systemic metastasis to the brain was seen by a visiting nurse at home (Fig. 1). The nurse found the patient with heart rate in the 40's without symptoms and was sent to the hospital for further management, where a 12 lead electrocardiogram demonstrated sinus tachycardia with complete heart block, junctional escape rhythm at 42 beats per minute and poor R wave progression in the precordial leads (Fig 2). An echocardiogram was obtained, which demonstrated normal left ventricular systolic function with concentric left ventricular hypertrophy. On echocardiography, the septum, inferior and posterior wall were thick and there was increased echogenicity of these walls which was thought to be from hypertrophy (Fig 3). Considering the history of systemic metastases of the malignant melanoma along with complete heart block, suspicion was raised to rule out cardiac metastasis. A gated cardiovascular magnetic resonance (CMR) study was performed. The steady state free precession (SSFP) cine vertical long axis and the horizontal long axis view of the left ventricle demonstrated normal left ventricular contractility with nodularity of the myocardium (Fig. 4A). This was representative of marked tumor infiltration of the myocardium (pan cardiac) with nodular deposits in the myocardial muscle layers with varying penetration into the endocardium and the epicardium. On SSFP cine sequences, these nodular deposits were discrete, isointense to bright compared to the normal myocardial muscle (Fig. 4A). The nodularity involved all myocardial walls including the membranous septum, proximal and distal anterior septum, inferior wall, anterior wall and the lateral wall (Fig. 4B). There were multiple mass-like deposits lining the right atrial and the left atrial wall with similar signal intensity as that of the left ventricular masses. The right ventricular free wall also demonstrated nodular deposits. On both T1 and T2-weighted images, the left and right ventricular and atrial masses were bright (Fig. 4B). On post-gadolinium images, these masses were enhancing due to contrast uptake and extracellular contrast retention. The cardiac valves appeared normal and there was minimal valvular disease (Fig. 4C and 4D). There was a small pericardial effusion, but the pericardial layer was free of disease. The hilar and paratracheal lymphadenopathy was present along with large left and small right pleural effusion.

## Discussion

Among all the malignancies metastasizing to the heart, malignant melanoma has the highest incidence (40% to 60% chance of cardiac metastasis in some series) [1-3]. The diagnosis of cardiac metastatic melanoma is usually incidental on antemortem diagnostic studies. The cardiac involvement of the metastasis is usually limited to the myocardium and pericardium; however, valvular and endocardial metastasis may be seen primarily in the right heart. There have been few case reports of conduction system involvement primarily by septal invasion of the tumor, but none describing the CMR characteristics of the extensive myocardial metastasis [4].

The gated CMR of the heart can characterize cardiac tumors into benign vs. malignant based on the signal characteristics of the T1-weighted; T2-weighted and post-gadolinium contrast enhancement of the myocardial masses [5]. Also, the cine sequences provide information about the chamber and valvular function in these cases. The T1 signal characteristics of malignant melanoma metastasis are dependent on the degree of melanin present in the tumor [6]. Most metastasis will be hyperintense on T1-weighted images due to high melanin content, which shortens the T1 relaxation time. The T2-weighted images are less influenced by melanin and are generally hyperintense due to increased proton density or water content of the tumor (Fig. 4B). The post-gadolinium images demonstrate enhancement due to increased vascularity and interstitial space between the disorganized anaplastic cells. There have been autopsy reports showing myocardial tissue replaced by extensive metastatic malignant melanoma nodular deposits appearing as pigmented epicardial melanotic (black) implants viewed on the surface of the heart by the naked eye, hence the name *charcoal heart *[7]. In our case, despite extensive myocardial replacement, the left ventricular systolic function was preserved. This intriguing finding was also reported by Waller BF et al [7].

## Conclusion

This case highlights the importance of surveillance and high suspicion of metastatic disease to the heart despite no cardiovascular clinical signs or symptoms. This case also underscores the importance of advanced diagnostic techniques such as CMR which can localize and characterize the metastasis. Most importantly, the CMR findings mimic the autopsy specimen of a similar case described by Waller BF et al, suggesting high pathological correlation. The cardiac presentation is often late and by that time the prognosis is ominous. Therefore a high suspicion of cardiac metastasis should be present and early referral and sequential follow-up with CMR should be encouraged.

Our patient underwent treatment with the investigational drug YM 155 in the past and more recently was on IL-2. She was started on prednisone and was receiving radiation treatment for intracranial metastasis. Due to her poor prognosis, a decision was reached between the treating physician and the patient to refrain from pacemaker treatment of complete heart block. She continued to be in intermittent complete heart block without cardiac symptoms when she was discharged from the hospital.

## Authors' contributions

VKR analyzed the images and drafted the manuscript. RBW and JY operated the MRI scanner, organized and stored the images. HG was involved in the treatment of the patient as well as review of the ECG and other diagnostic modalities. RWWB participated in scientific input. All authors read and approved the final manuscript.

## Consent

Written informed consent was obtained from the patient for publication of this Case report and accompanying photographs and images. A copy of the written consent is available for review by the Editor-in-Chief of this journal.

## References

[B1] Savoia P, Fierro MT, Zaccagna A, Bernengo MG (2000). Metastatic melanoma of the heart. J Surg Oncol.

[B2] Gibbs P, Cebon JS, Calafiore P, Robinson WA Cardiac metastases from malignant melanoma. Cancer.

[B3] Maude Tesolin MD, Chantale Lapierre MD, Luc Oligny MD, Jean-Luc Bigras MD, Martin Champagne MD (2005). Cardiac metastases from melanoma. RadioGraphics.

[B4] Ozyuncu N, Sahin M, Altin T, Karaoguz R, Guldal M, Akyurek O (2006). Cardiac metastasis of malignant melanoma: a rare cause of complete atrioventricular block. Europace.

[B5] Mousseaux E, Meunier P, Azancott S, Dubayle P, Gaux JC (1998). Cardiac metastatic melanoma investigated by magnetic resonance imaging. Magn Reson Imaging.

[B6] Premkumar A, Marincola F, Taubenberger J, Chow C, Venzon D, Schwartzentruber D (1996). Metastatic melanoma: correlation of MRI characteristics and histopathology. J Magn Reson Imaging.

[B7] Waller BF, Gottdiener JS, Virmani R, Roberts WC (1980). The "charcoal heart;" melanoma to the cor. Chest.

